# Application of Near-Infrared Spectroscopy for Evidence-Based Psychotherapy

**DOI:** 10.3389/fpsyg.2021.527335

**Published:** 2021-07-23

**Authors:** Sachiyo Ozawa

**Affiliations:** UTokyo Center for Integrative Science of Human Behavior (CiSHuB), Center for Evolutionary Cognitive Sciences, Graduate School of Arts and Sciences, The University of Tokyo, Tokyo, Japan

**Keywords:** near-infrared spectroscopy, evidence-based practice, psychotherapy, assessment, clinical psychology, neuroimaging

## Abstract

This perspective article discusses the importance of evidence-based psychotherapy and highlights the usefulness of near-infrared spectroscopy (NIRS) in assessing the effects of psychotherapeutic interventions as a future direction of clinical psychology. NIRS is a safe and non-invasive neuroimaging technique that can be implemented in a clinical setting to measure brain activity via a simple procedure. This article discusses the possible benefits and challenges of applying NIRS for this purpose, and the available methodology based on previous studies that used NIRS to evaluate psychotherapeutic effects. Furthermore, this perspective article suggests alternative methodologies that may be useful, namely, the single- and multi-session evaluations using immediate pre- and post-intervention measurements. These methods can be used to evaluate state changes in brain activity, which can be derived from a single session of psychotherapeutic interventions. This article provides a conceptual schema important in actualizing NIRS application for evidence-base psychotherapy.

## Introduction

### Evidence-Based Psychotherapy

Evidence-based practice in psychology (EBPP), which is defined by the American Psychological Association as “the integration of the best available research with clinical expertise in the context of patient characteristics, culture, and preferences” (American Psychological Association Presidential Task Force on Evidence-Based Practice, 2006), has been receiving increasing attention. EBPP aims to facilitate effective psychological practice based on empirically supported clinical principles. Although it includes a broad range of psychological practices (e.g., assessment, case formulation, psychotherapeutic relationships, and interventions), this article discusses the merits and challenges specifically in the context of an evidence-based approach to psychotherapy.

Throughout history, there has been a long-standing debate regarding the empirical evidence of psychotherapeutic effects (Chambless and Ollendick, 2001; Lilienfeld, 2007; Kazdin, 2008; Gaudiano and Miller, 2013; Emmelkamp et al., 2014; Cook et al., 2017). It could be misleading to depend on subjective interpretations and heuristic decisions with respect to psychotherapeutic events or effects; rather, the importance of scientific perspectives and empirically supported decision-making for psychotherapy implementation has been emphasized. In this regard, research has also provided ample evidence on the efficacy or effectiveness of various psychotherapeutic interventions, although most of these studies have focused on cognitive-behavioral therapy (e.g., Butler et al., 2006; Hofmann S.G. et al., 2012).

Various research methods are currently available to examine psychotherapeutic effects. Among them, self-report measures (e.g., questionnaires and interviews) and behavioral tasks (e.g., cognitive tests) are most frequently used in research and routine clinical practice. These methods are convenient to use and have provided substantial evidence for the effects of various psychotherapeutic modalities, when used properly. Contrarily, neuroimaging is being increasingly used for the assessment of psychotherapeutic effects in general research. Its reliability and validity are not sufficient compared with conventional methods, and its clinical use is often a target of debate. However, since psychiatric symptoms are associated with structural and/or functional abnormalities, and because psychotherapeutic interventions could lead to detectable structural and/or functional changes, neuroimaging has gained attention as another tool for assessing psychotherapeutic effects (Etkin et al., 2005; Roffman et al., 2005; Linden, 2008; Barsaglini et al., 2014). The information obtained from neuroimaging assessments has been expected to elucidate the mechanisms involved in disease pathogenesis, to monitor psychotherapeutic effects, and predict follow-up results (Etkin et al., 2005; Roffman et al., 2005; Barsaglini et al., 2014). Since neuroimaging is a biology-based technique, it evaluates different aspects from those assessed by conventional methods, and is thus expected to provide additional information.

### Aims of This Perspective Article

Previous research on psychotherapeutic effect evaluation using neuroimaging indices was mostly based on functional magnetic resonance imaging (fMRI) and positron emission tomography (PET). These neuroimaging techniques have high spatial resolution and are suitable in basic research for evaluating whole brain areas and neural mechanisms. However, their use for evidence-based psychotherapy is still limited partly because the preparation and procedure required to evaluate ongoing psychotherapy effects in a clinical setting may not be easily incorporated. Further, using these methods is expensive, which may impede the obtention of repeated measurements for detailed information of psychotherapeutic effects, although psychotherapeutic sessions often occur several times over the span of weeks or months.

Near-infrared spectroscopy (NIRS), which is a well-known, safe, and non-invasive functional neuroimaging technique, allows for assessment of brain activity using a simple procedure. Some NIRS systems are compact and portable and can be easily installed in a clinical setting. Portable systems usually require only a few minutes for attachment and preparation. Once NIRS systems are acquired, there is typically no additional cost for each measurement, which is useful for repeated measurements. Moreover, NIRS systems are relatively tolerant to body and head movements, allowing participants to comfortably sit on a chair during NIRS measurements. Therefore, the use of NIRS is suitable for a broad range of participants, including children and patients who have difficulty remaining immobile, such as those with attention-deficit hyperactivity disorder (ADHD) (Yasumura et al., 2014).

Given these characteristics, NIRS has been used for on-site measurements in various environments and applications, and researchers have suggested that NIRS systems can be useful tools in clinical psychology (Roffman et al., 2005; Adorni et al., 2016). However, their potential in the field of psychotherapy has not been fully realized yet. Despite psychotherapy being effective in clinical practice, there are many gaps regarding its effects and mechanisms. Through its unique characteristics, NIRS may be useful for providing additional or detailed information concerning these aspects. However, the methodology for examining psychotherapeutic effects using NIRS is not well organized. Therefore, this article discusses some of the possible benefits and challenges of applying this technique in psychotherapy and the available methodologies based on previous studies that have used NIRS to examine psychotherapeutic effects. Further, this perspective article proposes a methodology that could be useful in future research. In this way, the aim of this article was to provide a conceptual schema of methodology that may help facilitate evidence-based psychotherapy and highlight future directions for clinical psychology.

This perspective article initially introduces basic NIRS system characteristics (see section “Basic characteristics of NIRS”) and subsequently focuses on its application (see section “Agendas for NIRS application”). Thereafter, future directions of clinical psychology in terms of the application of NIRS for evidence-based psychotherapy are discussed in the following sections: “Methodology for evaluation of psychotherapeutic effects using NIRS,” “NIRS index for evaluation of psychotherapeutic effects,” and “Considerations for the use of NIRS as a practical clinical instrument.” Furthermore, the limitations and possibilities associated with the use of NIRS for evidence-based psychotherapy are discussed (see section “Limitations and possibilities”). This article conforms to the Declaration of Helsinki and was approved by the University of Tokyo (approval no. 694).

## Characteristics of NIRS and Agendas for NIRS Application

### Basic Characteristics of NIRS

Near-infrared spectroscopy monitors changes in hemoglobin oxygenation states by employing near-infrared light ranging from approximately 700 to 900 nm. Near-infrared light within this range is transparent when passing through body tissues, but is absorbed by hemoglobin in the blood, which attenuates the light. Cerebral blood flow (CBF) increases upon a regional and transient neural activation, a phenomenon known as neurovascular coupling (Roy and Sherrington, 1890). This leads to an increase in oxygenated hemoglobin (oxyHb) levels and a simultaneous decrease in deoxygenated hemoglobin (deoxyHb) levels (Obrig et al., 1996), which affect the amount of light attenuation. Light attenuation is measured, using emitters and detectors that are attached to the head surface, and converted to changes in concentrations of oxyHb and deoxyHb using the modified Beer-Lambert law (Delpy et al., 1988). The concentration changes correspond to the amount of brain activity in that particular region.

**FIGURE 1:**
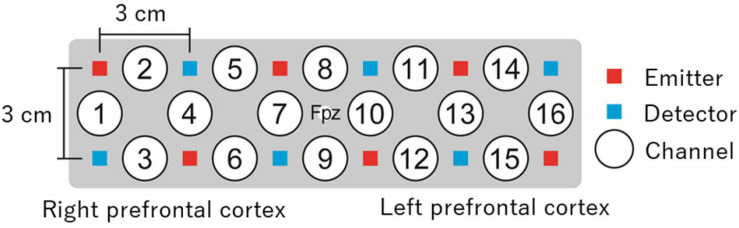
An example of prefrontal measurement with 16-channel NIRS. In this example, a holder is placed on the head surface such that its center matches the Fpz location in the international 10–20 system. Changes in oxyHb and deoxyHb concentrations are measured from approximately 2 cm below the scalp by 16 independent channels. NIRS, near-infrared spectroscopy; oxyHb, oxygenated hemoglobin; deoxyHb, deoxygenated hemoglobin.

The measurement methods may differ depending on the NIRS system. However, in most systems, light emitters and detectors, both known as optodes, are attached at specific positions on the head surface based on internationally standardized methods for electroencephalography electrode positioning (e.g., the 10–20 system) (Jasper, 1958). The optodes are usually fixed 3 cm apart from each other using a holder or cap (Figure 1). Changes in oxyHb and deoxyHb concentrations are measured at each channel located in the middle of an emitter and detector, approximately 2 cm below the scalp. The number and locations of measurable channels differ depending on the NIRS system. Various types of NIRS systems, including portable systems and systems equipped with one or several channels, are available.

### Agendas for NIRS Application

To use NIRS for the evaluation of psychotherapeutic effects, several considerations must be taken into account; while certain challenges may emerge during actualization and will need to be addressed. In this section, some of these considerations are discussed.

Because near-infrared light reaches a maximum depth of approximately 2 cm below the head surface, the areas of the brain that can be studied with NIRS are limited. NIRS is usually only applicable for surface cortical regions, although variations in cortical thickness may generate discrepancies depending on the brain region and individuals (Okamoto et al., 2004). This limitation restricts the types of research questions that can be investigated using NIRS. Nevertheless, the measurable areas cover the prefrontal cortex, which engages in essential higher cognitive functions (Smith and Jonides, 1999), such as executive functions that include updating (ability to monitor information and update working memory representations), inhibition (ability to inhibit prominent responses), and shifting (ability to shift mental sets) (Miyake et al., 2000; Alvarez and Emory, 2006; Friedman et al., 2008; Minzenberg et al., 2009; Best and Miller, 2010; Hofmann W. et al., 2012). These functions are critical for understanding the dysfunctions associated with psychiatric disorders, as will be discussed later. Moreover, because a particular region of the brain is associated with multiple functions, different brain activities can be observed in the same area using an experimental control. For example, the dorsolateral prefrontal cortex (DLPFC) engages in executive functions, as well as participates in decision making (Krain et al., 2006) and social cognitions such as theory of mind (Kalbe et al., 2010). Thus, despite the area limitation, NIRS can be used to assess various important functions using an appropriate experimental control.

The fact that a particular brain region involves multiple functions indicates that it may be challenging to distinguish brain functions based on regional CBF (rCBF) changes using NIRS alone. For example, the medial prefrontal cortex (MPFC) regions were found to be involved in both regulation of emotion (brain activity for emotion regulation, such as appraisal and suppression; Diekhof et al., 2011; Nelson et al., 2015) and responses to emotional material (brain activity for responses to emotional stimulation; Phan et al., 2002; Ochsner et al., 2009). Therefore, rCBF changes in the MPFC could be interpreted due to either function. Nevertheless, underlying functions related to rCBF changes need to be identified without information from deep brain regions (e.g., the limbic system, which constitutes the center of emotional processing), using NIRS alone (Ozawa et al., 2014, 2019; Ozawa and Hiraki, 2017). To address these concerns, adequate basic research is required before the application of NIRS in clinical practice. Complementary use of other techniques, such as fMRI (Kirilina et al., 2012; Sato et al., 2013; Haeussinger et al., 2014) and physiological measurements (Balconi et al., 2015; Pinti et al., 2015), can be beneficial.

Finally, NIRS signals are easily affected by the activity of peripheral physiological processes controlled by the autonomic nervous system (e.g., heart rate, respiration, and blood pressure) (Kirilina et al., 2012; Haeussinger et al., 2014) that involve the continuous contraction and relaxation of innervated blood vessels (Söderstrom et al., 2003). In particular, task-evoked physiological changes in extracerebral blood vessels, such as changes in skin blood flow, cause artifacts in NIRS signals (Takahashi et al., 2011; Kirilina et al., 2012). For example, artifacts are frequently observed during tasks that affect respiration (e.g., verbal fluency tasks) (Takahashi et al., 2011), emotional processing (e.g., picture presentation) (Minati et al., 2009), and body movement (e.g., arm raising) (Minati et al., 2011). These risks may be reduced by contriving experimental designs. Performing tasks during baseline instead of resting has been suggested to examine intended differences by reducing possible confounding factors (Minagawa et al., 2017). The risks may be further reduced by controlling factors that cause artifacts. For example, performing adequate preliminary practices or creating a relaxed resting state at the beginning (using deep breathing or attentional control, for example) may promote stabilization of arousal. Body movement during tasks can be reduced using body supports such as a chin or arm rest. Furthermore, various analytical methods have been developed to reduce physiological artifacts (Scholkmann et al., 2014; Tak and Ye, 2014), including independent component analysis (Kohno et al., 2007), spatial filtering (Zhang et al., 2016), and the hemodynamic modality separation method (Yamada et al., 2012). For the hemodynamic modality separation method, a free data analysis package is currently available (The National Institute of Advanced Industrial Science and Technology, 2019).

## Future Directions in Clinical Psychology

### Methodology for Evaluation of Psychotherapeutic Effects Using NIRS

There are several possible methods for evaluating psychotherapeutic effects using NIRS systems (Tables 1, 2). One is to measure brain activity during a psychotherapeutic intervention or a similar condition. This allows for detection of the neural mechanisms underlying the intervention through real-time monitoring during the intervention. This method is particularly suitable with NIRS and has been used in various forms of psychotherapy (Tables 1, 2), such as during mindfulness meditation (Gundel et al., 2018; Rosenbaum et al., 2020a), interpersonal brain synchronization between the counselor and client (Zhang et al., 2018, 2020), attention training with metacognitive therapy (Rosenbaum et al., 2018), exposure to stimuli for specific phobia (Landowska et al., 2018; Rosenbaum et al., 2020b), role lettering in writing therapy (Okamoto et al., 2010), and trauma-related recall with eye movement desensitization (Ohtani et al., 2009; Amano and Toichi, 2016). Nevertheless, this method restricts body movement during the intervention, which could disrupt performance, depending on the type of psychotherapy. Hence, this method has limited applicability for psychotherapeutic effect evaluation.

Second, psychotherapeutic effects can be assessed by comparing pre-intervention rCBF changes with post-intervention rCBF changes during task implementation, thereby detecting changes in brain function over a series of sessions. This method has typically been used for the examination of empirical evidence of psychotherapeutic effects, often employing structurally established experimental designs (including randomized controlled trials). For neuroimaging modalities, fMRI (Haut et al., 2010; Bor et al., 2011; Yoshimura et al., 2014) is frequently used; however, it is also used with NIRS (Tables 1, 2). For example, the effects of the neuropsychological educational approach to cognitive remediation were assessed by pre- and post-comparison of the oxyHb changes during 2-back tasks (Pu et al., 2014). Similarly, the effects of haptic-assisted meditation were evaluated using the attention network and sustained attention to response tasks (Zheng et al., 2019); further, the effects of acceptance and commitment therapy were assessed using behavioral tasks including editing, mirror image tracking, and circle tracking (Ong et al., 2020).

**TABLE 1:** Experimental designs of studies that evaluated psychotherapeutic effects using hemodynamic response changes.

Study	Target participants	*N* (male)	Age	Psychotherapy	Session periods	Contrasts (based on findings)	Design	Randomization	Real-time monitoring
Gundel et al., 2018	Meditation experts	14 (6), 16 (6)	49.2, 22.5	Mindfulness	1 session	Groups with (EXP) vs. without (CON) meditation experiences	Between	–	Yes
Rosenbaum et al., 2020a	Healthy participants	16 (4), 18 (3) for EXP, 17 (5), 16 (7) for CON	16 (4), 18 (3) for EXP, 17 (5), 16 (7) for CON	Mindfulness	1 session	Mindfulness instruction (EXP) vs. instruction thinking (CON) groups; focus vs. equanimity conditions in EXP	Between/within	Yes	Yes
Zhang et al., 2018	Healthy participants (students as clients)	34 (5) total	21.1	Psychological counseling	1 session (40 min)	Counseling (EXP) vs. chatting (CON) groups	Between	Yes	Yes (hyper scanning)
Zhang et al., 2020	Healthy participants (students as clients)	14 (0), 16 (0)	21.1	Psychological counseling	1 session (40 min)	Experienced (EXP) vs. novice (CON) counselor groups	Between	Yes	Yes (hyper scanning)
Rosenbaum et al., 2018	Healthy participants	46 (18)	24	Attention training techniques (ATT; metacognitive therapy)	1 session	Three attention training (EXP) conditions vs. resting (CON) condition	Within	–	Yes
Zheng et al., 2019	Healthy participants	10 (6)	23.1	Haptics-assisted meditation	5 sessions	Pre vs. post intervention; haptic-assisted meditation (EXP) vs. rest (CON) conditions	Within (for NIRS measurement)	–	Yes
Landowska et al., 2018	Participants with a fear of heights	14 (2)	42.3	Virtual reality exposure therapy	3 sessions (each session includes pit and training conditions)	Pit (EXP) vs. training (CON) room condition; first vs. third sessions	Within	–	Yes
Okamoto et al., 2010	Healthy participants	16 (13)	12.1	Role lettering (writing therapy)	Once a week for 12 months	Pre vs. 3 vs. 6 vs. 12 months for the retrograde writing task; pre vs. 3 vs. 6 vs. 12 months for the antegrade writing task	Within	–	Yes
Rosenbaum et al., 2020b	Specific phobia/arachnophobia	37 (4), 7 dropouts	28.7	Exposure therapy	5 sessions	Film clips of spiders (EXP) vs. house animals (CON) condition; beginning vs. end sessions	Within	Yes (cross-over design)	Yes
Amano and Toichi, 2016	Healthy participants	7 (3)	34.4	Eye movement desensitization and reprocessing (EMDR)	1 session	Four condition comparison: trauma-related recall only (EXP1), trauma-related recall with EM (EXP2), cognitive modification (EXP3), rest (CON)	Within	–	Yes
Ohtani et al., 2009	PTSD	13 (3)	33	Eye movement desensitization and reprocessing (EMDR)	1 session a week across 2–10 weeks	Four condition comparison: trauma-related recall only (EXP1), eye movement (EM) only (EXP2), trauma-related recall with EM (EXP3), no recall nor EM (CON); Pre vs. post intervention	Within	–	Yes
Pu et al., 2014	Schizophrenia or schizoaffective disorder	19 (11), 12 (9)	28.5, 31.4	Neuropsychological educational approach to cognitive remediation (NEAR)	2 sessions a week for 6 months	NEAR (EXP) vs. no cognitive training (CON) groups; pre vs. post intervention	Between/within	No	No
Ong et al., 2020	Participants with clinical perfectionism	14 (3), 15 (7)	30.4, 23.1	Acceptance and commitment therapy (ACT)	10 sessions	ACT (EXP) vs. waitlist (CON) groups, pre vs. post intervention	Between/within	Yes	No
Glassman et al., 2016	SAD (public speaking anxiety)	11, 10 (including 24% males)	29.9, 26.1	Two types of brief cognitive-behavioral interventions	1 session (90 min)	Traditional cognitive-behavior treatment (tCBT) vs. acceptance-based behavior treatment (ABBT) groups; pre vs. post intervention	Between/within	Yes	No
Izzetoglu et al., 2020	Healthy participants	21 (10)	20.5	Loving-kindness meditation	1 session	Meditation (EXP) vs. Stroop color word task (CON); Pre vs. post intervention	Within	–	Yes

*The list shows the results of an online search of the PubMed and Web of Science databases. Search terms = (“NIRS” OR “near-infrared spectroscopy”) AND (“psychotherapy” OR “psychological therapy” OR “psychological intervention” OR “psychological treatment” OR “CBT” OR “cognitive-behavioral therapy” OR “mindfulness” OR “interpersonal therapy” OR “psychodynamic therapy” OR “acceptance and commitment therapy” OR “remediation therapy” OR “cognitive remediation” OR “social skill training”). The search was performed on February 23, 2021. No time span was specified. The search obtained 145 hits from PubMed and 44 hits from Web of Science. Studies that met the following criteria were excluded: (1) reviews, proceeding/conference papers, sections of books, and protocols, (2) not psychotherapy (e.g., medication, repetitive transcranial magnetic stimulation, transcranial direct-current stimulation, mathematic learning for education), (3) use of NIRS as therapeutic techniques (e.g., NIRS-neurorehabilitation, Brain computer interface), (4) not aimed for intervention on psychiatric symptoms (e.g., neurological symptoms, physical pains or movements), (5) not aimed to examine effects of interventions (e.g., examination of neural mechanisms for symptoms or task performance, or biomarkers, having no experimental contrasts between certain psychotherapeutic conditions). Consequently, the list includes only peer-reviewed articles that examined psychotherapeutic effects using NIRS. Information most relevant for this article was extracted from the studies.*

*CON, control group or condition; EM, eye movement; EXP, experimental group or condition; PTSD, post-traumatic stress disorder; SAD, social anxiety disorder.*

**TABLE 2:** Brain and behavioral results of studies that evaluated psychotherapeutic effects using hemodynamic response changes.

Study	NIRS devise	Measurements of brain activity	Activation contrasts	Activation regions for the contrasts	Index changes	Index	*p*-value	Behavioral or symptomatic changes	Brain-behavioral or demographic data correlation
Gundel et al., 2018	44 CHs (two lateral probe-sets) (ETG-4000)	Mindfulness task (BL and mindfulness conditions)	EXP > CON (in mindfulness condition)	Right auditory cortex (BA 1, 6, and 40)	↓	HHb	*p* = 0.048 to 0.031	–	–
Rosenbaum et al., 2020a	46 CHs (two frontal and one parietal probe-sets) (ETG-4000)	Mindfulness task (focus and equanimity conditions) and emotion regulation task	EXP < CON for mindfulness task; focus < equanimity in EXP; EXP > CON for emotion regulation task	CCN; lDLPFC, the left inferior prefrontal gyrus, and superior parietal lobule; lDLPFC	↓; ↓ (reduced distress); ↑	O_2_Hb	*p* < 0.1 (trend); *p* < 0.05 to 0.001; *p* < 0.05	Emotional distress↓	–
Zhang et al., 2018	22 CHs (the PFC probe-set) and 24 CHs (the right temporo-parietal probe-set) (ETG-7100)	IBS during counseling and chatting	EXP > CON (during period of 40∼44 s)	IBS of the right temporo-parietal junction	↑	O_2_Hb	*p* < 0.001	Self-rated alliance↑ Total scores and the goal subscales in WAI-SR↑	The right temporo-parietal junction↑ and the affective bond subscale in WAI-SR↑ (*r* = 0.55, *p* = 0.023) in EXP
Zhang et al., 2020	24 CHs (the right temporo-parietal probe-set) (ETG-7100)	Interpersonal brain synchronization (IBS) during counseling	EXP > CON (during period of 22∼42 s)	IBS of the right temporo-parietal junction	↑	O_2_Hb	*p* < 0.05 to 0.01	Total scores and the task and goal subscales in WAI-SR↑	The right temporo-parietal junction↑ and the goal subscale in WAI-SR↑ (*r* = 0.54, *p* = 0.032) in EXP
Rosenbaum et al., 2018	46 CHs (two frontal and one parietal probe-sets) (ETG-4000)	Attention training paradigm	EXP > CON	The right inferior frontal gyrus, somatosensory association cortex, rDLPFC	↑	O_2_Hb	*p* < 0.05	Subjective effort↑ in EXP	The right inferior PFC↑ and subjective effort↑ (*p* < 0.05)
Zheng et al., 2019	64 CHs (prefrontal and sensorimotor regions) (NirScan)	Attention network task (ANT), sustained attention to response task (SART), and haptic-assisted meditation	Pre < post; EXP > CON	PFC and bilateral sensorimotor regions in ANT, rPFC in SART; bilateral PFC and sensorimotor regions	↑; ↑	O_2_Hb	*p* = 0.032 to 0.003; *p* = 0.028 to 0.001	Attentional performance↑	–
Landowska et al., 2018	20 CHs (PFC region) (NIRSport)	Stimuli of a virtual pit or training room	EXP > CON in third session; first < third sessions	Bilateral DLPFC and bilateral medial PFC; lDLPFC	↑;↑	O_2_Hb	*p* < 0.05 (corrected); *p* < 0.05 (corrected)	SUDS↓	Right medial PFC↑ and anxiety related to height↓ (*r* = –0.603 to –0.650, *p* = 0.029 to 0.016)
Okamoto et al., 2010	22 CHs (frontal probe-set) and 24 CHs (bilateral probe-sets) (ETG-4000)	Retrograde and antegrade writing tasks	Pre > 6 and 12 months later in retrograde task; *n.s.* in antegrade writing task	Right lateral region	↓ (reduced emotion); *n.s.*	O_2_Hb	*p* < 0.001	Number of words 12 months later↑ in both tasks	Right lateral region↓ and number of words 12 months later↑ (*r* = –0.0318, *p* < 0.05 in retrograde task; *r* = –0.344, *p* < 0.01 in antegrade task)
Rosenbaum et al., 2020b	46 CHs (two frontal and one parietal probe-sets) (ETG-4000)	Film clips of spiders and house animals	Beginning > end sessions during exposure phase	lDLPFC, the left inferior prefrontal gyrus, and superior parietal lobule (CCN related regions)	↓ (reduced need for control)	O_2_Hb	*p* < 0.05	BAT↓SPQ↓SBQ↓FSQ↓	Higher correlation between CCN and anxiety decreased within following sessions.
Amano and Toichi, 2016	52 CHs (ETG-4000)	EMDR paradigm	EXP2 < EXP1	The right superior temporal sulcus	↓ (reduced need for control)	O_2_Hb	*p* = 0.044	POMS↓ SUDS↓ VOC↑	–
Ohtani et al., 2009	24 CHs (left and right PFC probe-sets) (ETG-100)	EMDR paradigm	EXP3 < EXP1; Pre > post in EXP1 and EXP3	LPFC	↓; ↓ (reduced need for control)	O_2_Hb	*p* = 0.001; *p* = 0.01	IES-R↓SUDS↓VOC↑CAPS↓	LPFC↓ and CAPS↓ (*r* = 0.66, *p* = 0.02)
Pu et al., 2014	52 CHs (ETG-4000)	2-back task (letter version)	Pre < post in EXP	Bilateral DLPFC (BA 9, 46), lVLPFC (BA45), rFPC (BA10)	↑	O_2_Hb	*p* = 0.05 to 0.005	2-back performance↑BACS-J↑PANSS↓	Verbal memory in BACS-J↑ and superior and medial temporal cortices (BA21,22) and the temporopolar area (BA38)↑ (ρ = 0.49 to 0.57, *p* < 0.05 to 0.01). Verbal fluency in BACS-J↑ and rDLPFC (BA21,22) and frontopolar (BA10)↑ (ρ = 0.47 to 0.61, *p* < 0.05 to 0.01).
Ong et al., 2020	44 CHs (two probe-sets for the PFC and the right side of head) (ETG-4000)	Behavioral tasks (editing, mirror image tracking, circle tracking)	Pre > post in EXP (greater reduction than in CON)	lDLPFC for all tasks, rDLPFC and dorsal medial PFC for editing, right inferior parietal lobe for circle tracking in EXP	↓ (reduced need for control)	THb	–	Clinical perfectionism↓ symptom distress and functional impairment↓ quality of life↑ progress toward valued living↑ psychological inflexibility↓ self-compassion↑	–
Glassman et al., 2016	16 CHs (PFC probe-set) (Izzetoglu et al., 2005)	Behavioral avoidance test speech	Pre > post in ABBT; pre < post in tCBT	lDLPFC in ABBT; lDLPFC in tCBT	↓ (reduced need for control); ↑	THb	(trend)	SUDS↓ observer-rated SPS↑ in both groups	–
Izzetoglu et al., 2020	4 CHs (lPFC and rPFC) (fNIR Imager)	Meditation and Stroop color word task	EXP > CON; Pre < post	lPFC and rPFC	↑;↑	O_2_Hb, HHb, THb	*p* < 0.05 with HHb and THb (trend with O2Hb); *p* < 0.05 with THb	Response time↓ blood pressure↓	–

*ABBT, acceptance-based behavior treatment; BA, brodmann area; BACS-J, brief assessment of cognition in schizophrenia, Japanese version; BAT, behavioral avoidance test; CAPS, clinician administered PTSD scale; CCN, cognitive control network; CHs, channels; CON, control group or condition; DLPFC, dorsolateral prefrontal cortex; EMDR, eye movement desensitization and reprocessing; EXP, experimental group or condition; FPC, frontal pole cortex; FSQ, fear of spider questionnaire; HHb, deoxygenated hemoglobin; IBS, interpersonal brain synchronization; IES-R, impact of event scale-revised; IQ, intelligence quotient; LPFC, lateral prefrontal cortex; O_2_Hb, oxygenated hemoglobin; PANSS, Positive and Negative Syndrome Scale; PFC, prefrontal cortex; POMS, profile of mood states; SBQ, Spider Beliefs Questionnaire; SPQ, Spider Phobic Questionnaire; SPS, Speech Performance Scale; SUDS, Subjective Units of Distress Scale; tCBT, traditional cognitive-behavior treatment; THb, total hemoglobin; VLPFC, ventrolateral prefrontal cortex. VOC, validity of cognition; WAI-SR, Working Alliance Inventory-Short Revised.*

**FIGURE 2:**
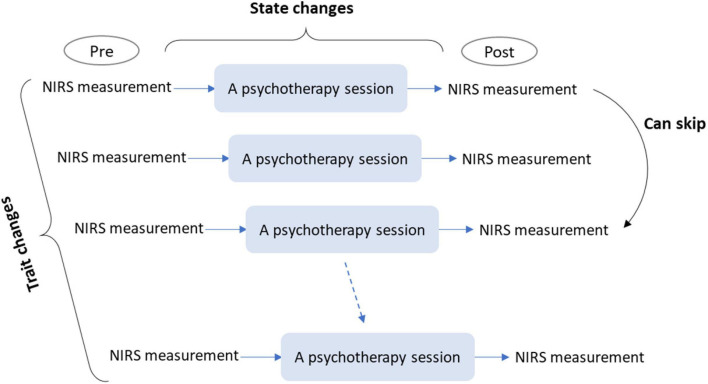
A conceptual schema for single- and multi-session evaluations of psychotherapeutic effects using NIRS. Single-session evaluation can be conducted by immediate pre- and post-intervention measurements of rCBF changes. This evaluation allows for the measurement of state changes in brain activity induced by a single session. Multi-session evaluation can be conducted by repetitive single-session evaluations, which allows for the examination of trait changes in brain activity induced by a psychotherapy program. NIRS, near-infrared spectroscopy; rCBF, regional cerebral blood flow.

Third, pre- and post-intervention measurements of rCBF changes could be interposed in a single session (Figure 2). Although the previous method measures trait changes derived from a series of sessions, this method measures state changes derived from a single session. In comparison with pre- and post-intervention measurements over a series of sessions, performing measurements in one session would have the following benefits:

• Physical and mental conditions of patients, which constantly vary, are less likely to influence the obtained signals.
• Immediate measurement will facilitate the capture of subtle changes induced by a single session.
• Determination of the effectiveness of a single session is possible.

Although this method has rarely been used, previous studies have utilized it to evaluate the effects of brief cognitive-behavioral interventions for public speaking anxiety (Glassman et al., 2016) and loving-kindness meditation in healthy participants (Izzetoglu et al., 2020). This method demands immediate and repetitive measurements, preferably performed on-site; therefore, it would be particularly suitable with NIRS. The present article suggests that the method is useful in the application of NIRS for evaluating psychotherapeutic effects.

Finally, pre- and post-intervention measurements in a single session can be extended to multi-sessions (Figure 2). This requires repetitive measurements over a series of sessions and would be feasible only with NIRS. It can facilitate the investigation of changes in effects over a series of sessions (e.g., whether initial or latter sessions were more effective). Since most psychotherapeutic approaches involve multiple sessions, this investigation would be beneficial for most approaches.

For single- and multi-session evaluations of psychotherapeutic effects using NIRS, immediate measurement is important since it measures state changes in brain activity, which is suitable with NIRS. To perform immediate measurements in a clinical setting, an easy and simple process for NIRS measurement is needed; the NIRS system should be attached quickly to the patient or client’s head. Furthermore, simple and short tasks are required; implementation of the task should not disrupt the psychotherapy session or become a burden for the patient or client.

### NIRS Index for Evaluation of Psychotherapeutic Effects

The NIRS index, which can be used to assess psychotherapeutic effects, should be carefully examined in future studies. Setting a certain norm which can be applied for broad psychiatric disorders or symptoms may be challenging since each disorder or symptom may have unique characteristics. However, the present article suggests the most likely candidates for NIRS indices which can be used to evaluate psychotherapeutic effects since certain tendencies have already been indicated in previous studies. The most likely candidate for a NIRS index would be the concentration changes of oxyHb in the lateral prefrontal cortex (LPFC) region, including the DLPFC, during executive tasks. LPFC activity is known to increase with the employment of executive functions, such as working memory, inhibition, and emotional control. Table 3 lists some previous findings regarding prefrontal activities during executive tasks in patients with psychiatric disorders, in contrast to healthy participants.

**TABLE 3:** OxyHb levels changes during executive tasks in psychiatric patients in comparison with healthy controls.

Study	Contrasts	*N* (male)	Age	Measurements of brain activity	Brain regions	oxyHb level change	*p*-value	Brain-behavioral or demographic data correlations
Ishii-Takahashi et al., 2014	ASD, ADHD < CON	21 (8), 19 (11), 21 (13)	30.8, 30.6, 28.8	Stop signal task	Broad PFC regions for ASD, the right premotor region, right presupplementary motor region, bilateral DLPFC for ADHD	↓	*p* < 0.01 to 0.001	No correlations with clinical symptoms, task performance, sex
Ueda et al., 2018	ADHD < CON	12 (4), 12 (4)	32.5, 32.1	Stroop color-word task	Inferior PFC	↓	*p* < 0.05, Bonferroni-corrected	oxyHb↓ and symptom severity↑ (CAARS-Inv; ρ = –0.614 to 0.639, *p* < 0.05) in ADHD
Xiao et al., 2012	Children with HFA, ADHD < CON	19 (19), 16 (16), 16 (16)	10.1, 9.8, 9.7	Go-no/go task (no-go block condition)	rPFC	↓	*p* = 0.046 to 0.009	–
Tsujimoto et al., 2013	Children with ADHD > CON	16 (16), 10 (10)	10.9, 10.1	Visuospatial working memory task (on distractor condition)	rPFC, MPFC	↑ (compensatory responses)	*p* = 0.03 (*d* = 0.94) for rPFC, *p* = 0.002 (*d* = 1.39) for MPFC	oxyHb↑ and error rate↑ (*r* = 0.55, *p* = 0.03 for rPFC; *r* = 0.58, *p* = 0.02 for MPFC) in ADHD
Yasumura et al., 2014	Children with ADHD < CON	10 (8), 15 (6)	11.2, 9.6	Reverse Stroop task	rLPFC	↓	*p* = 0.033	oxyHb↓ and inattention score↑ (*r* = –0.6, *p* < 0.068) in ADHD (trend)
Suzuki et al., 2017	Children with ADHD > CON	12 (11), 14 (10)	9.9, 9.5	Flanker task	The left superior PFC	↑ (proactive control)	*p* = 0.01	oxyHb↑ and inattention score↑ (*r* = 0.58, *p* < 0.05) in ADHD
Kaga et al., 2020	Children with ADHD < CON	20 (17), 18 (11)	9.9, 10.2	Colored go-no/go task	rPFC	↓	*p* < 0.05 to 0.01	oxyHb↓ and scores of Stroop test↓ (*r* = 0.376 to 0.457, *p* = 0.045 to 0.014), inattention↓ (*r* = 0.376, *p* = 0.045)
Ikeda et al., 2018	Children with ASD < CON	24 (17), 24 (18)	10.0, 9.6	Go-no/go task	The right inferior frontal gyrus/middle frontal gyrus	↓	*p* < 0.05 (*d* = 0.61)	–
Ikeda et al., 2013	MDD < CON; MDD > CON	MDD, 21 (7); CON, 16 (7)	40.9, 36.2	Verbal fluency test; Stroop task (on the incongruent condition)	Bilateral PFC in verval bluency task; bilateral PFC in Stroop task (in the incongruent condition)	↓; ↑ (impaired selective attention)	*p* = 0.037; *p* = 0.039	–
Uemura et al., 2014	Erderly with depression < CON	13 (6), 67 (28)	74.5, 73.8	Trail making test-B	Bilateral PFC	↓	*p* = 0.03	oxyHb↓ and depressive symptoms↑ (*r* = –0.33, *p* = 0.003 for lPFC; *r* = –0.27, *p* = 0.02 for rPFC), education↓ (*r* = 0.27, *p* = 0.01 for lPFC; *r* = 0.33, *p* = 0.03 for rPFC), age↑ (*r* = –0.37, *p* = 0.01 for lPFC; *r* = –0.33, *p* = 0.003 for rPFC), or number of medication↑ (*r* = –0.24, *p* = 0.04 for lPFC; *r* = –0.25, *p* = 0.02 for rPFC)
Ohtani et al., 2015	BD, MDD < CON	18 (9), 10 (4), 14 (7)	39.7, 39.2, 33.6	Verbal fluency test	Bilateral VLPFC, the anterior part of the temporal cortex	↓	*p* < 0.001	No correlations with VFT performance, medication dosage, HAM-D, YMRS, SASS, age, education, estimated IQ
Nishimura et al., 2015	BD hypomanic, BP depressed < CON	11 (8), 16 (10), 12 (4)	44.0, 44.6, 46.4	Verbal fluency test	Bilateral PFC	↓	*p* < 0.05, FDR-corrected	oxyHb↓ and YMRS↓ (ρ = 0.660 to 0.727, FDR-corrected *p* < 0.05), doses of medication↓↑ (ρ: –0.898 to 0.660, *p* < 0.05)
Ono et al., 2017	Adolescents with BDII > CON	10 (3), 10 (2)	15.7, 15.0	Verbal fluency test (during the early phase)	Bilateral Inferior PFC	↑ (compensatory responses)	*p* = 0.0133 for the left, *p* = 0.0079 for the right	–
Fu et al., 2018	BP < CON; BP < CON	43 (17), 32 (15)	24.7, 26.7	Tower of London task; Verbal fluency test	Bilateral DLPFC in Tower of London task; rVLPFC, rDLPFC, rPFC, lPFC in verbal fluency task	↓; ↓	*p* = 0.049 to 0.041 (*p* < 0.05, FDR-corrected); *p* = 0.046 to 0.027 (*p* < 0.05, FDR-corrected)	oxyHb↓ and HAMD↑ (*r* = –0.460, *p* = 0.002) in verbal fluency task
Kiriyama et al., 2020	MDD < CON	18 (12), 22 (11)	44.2, 42.0	Verbal fluency test	Bilateral PFC, temporal regions	↓	*p* = 0.0137 to.0006, FDR-corrected	oxyHb↓ in the left temporal regions and motor speed↑ in BACS-J (ρ = 0.904, *p* < 0.0001, FDR-corrected) in MDD
Koike et al., 2011	ChSZ < FEP < UHR < CON	38 (22), 27 (18), 22 (13), 30 (17)	31.3, 25.2, 21.6, 24.3	Letter fluency task	Bilateral DLPFC	↓	*p* < 0.05, FDR-corrected	oxyHb↑ and PANAS positive scores↑ (*r* = 0.615, FDR-corrected, *p* = 0.001), oxyHb↑ and PANAS negative scores↑ (*r* = 0.606 to 0.759, FDR-corrected *p* = 0.001) in FEP (compensatory responses)
Fujiki et al., 2013	SCZ < CON	14 (5)	29.9, 30.9	Trail making test -A and -B	FPC and midfrontal regions	↓	*p* < 0.01 in test-A and -B	oxyHb↓ and test-A performance↓ (*r* = 0.57, *p* < 0.05), oxyHb↓ for test-A and PANAS positive scores↑ (*r* = –0.56 to –0.70, *p* < 0.01), oxyHb↓ for test -B and PANAS positive scores↑ (*r* = –0.60 to –0.81, *p* < 0.01)
Pu et al., 2013	SCZ < CON	30 (21), 30 (19)	32.1, 32.4	Verbal fluency test	PFC and temporal regions	↓	*p* < 0.045 to 0.001, FDR-corrected	oxyHb↓ in rVLPFC and temporal regions and self-reflectiveness in BCIS↓ (*r* = 0.52 to 0.64, *p* < 0.005 to.001); no correlations with self-certainty in BCIS, task performance, age, duration of illness, PANAS scores, daily dosage of antipsychotic drugs
Pu et al., 2016	SCZ < CON	26 (18), 26 (18)	31.6, 31.2	2-back task	LPFC, FPC, temporal regions	↓	*p* < 0.05, FDR-corrected	oxyHb↓ in LPFC, FPC and temporal regions and SCSQ ToM subscale↓(ρ = 0.475 to 0.782, *p* < 0.05, FDR-corrected); no correlations with other subscales in SCSQ
Pu et al., 2017	SCZ < CON	23 (16), 23 (16)	31.8, 31.0	2-back task	VLPFC, the anterior part of the temporal cortex, DLPFC, FPC	↓	*P* < 0.05 to 0.001	oxyHb↓ and SCSQ ToM subscale↓ (*r* = 0.463, *p* < 0.05)
Nakano et al., 2018	SCZ < CON	28 (14), 28 (14)	30.8, 30.8	Tree-drawing task	Left middle frontal region, bilateral inferior PFC, bilateral inferior parietal regions, left superior temporal regions	↓	*P* < 0.01 to 0.001	oxyHb↑ and PANAS positive scores↑ (*r* = 0.410, *p* = 0.030) or PANAS negative scores↑ (*r* = 0.406, *p* = 0.032) for free-drawing task (compensatory responses); oxyHb↓ and PANAS positive scores↑ (*r* = –0.482, *p* = 0.009) or PANAS negative scores↑ (*r* = –0.392, *p* = 0.039) for copying task
Pu et al., 2019	ASD, SCZ < CON	32 (25), 87 (37), 50 (20)	28.0, 33.6, 34.4	2-back task	lDLPFC, lFPC	↓	*p* < 0.004, FDR-corrected, for ASD; *p* < 0.036, FDR-corrected, for SCZ	oxyHb↓ and BACS-J↓ (ρ = 0.205 to 0.447, *p* < 0.031, FDR-corrected)
Gündüz et al., 2020	SCZ < CON	20, 23	34.6, 30.0	Episodic future thinking	PFC	↓	*p* < 0.01	oxyHb↑ in rPFC and performance in future imagination with 3 given cues↓ (*p* < 0.01) in SCZ (interferences of stimulus independent thoughts)
Roberts and Montgomery, 2015	Ecstasy polydrug users > CON	20 (13), 20 (8)	21.9, 20.9	Chicago word fluency test (oral variant)	lDLPFC, rMPFC	↑ (compensatory responses)	*p* < 0.05 to 0.01	–

*The list shows the results of an online search of the PubMed and Web of Science databases. Search terms = (“NIRS” OR “near-infrared spectroscopy”) AND (“executive function” OR “cognitive control”) AND (“psychopathology” OR “psychiatric” OR “schizophrenia” OR “schizophrenic” OR “depression” OR “depressed” OR “ADHD” OR “anxiety” OR “PTSD”). The search was performed on February 23, 2021. No time span was specified. The search obtained 32 hits from PubMed and 69 hits from Web of Science. From the search results, only peer-reviewed articles that involve an experimental study regarding brain activities of executive functions of psychiatric patients using NIRS, in comparison with healthy participants, were selected and shown in the list. Information most relevant for this article was extracted from the studies. ADHD, attention-deficit hyperactivity disorder; ASD, autism spectrum disorder; BACS-J, Japanese version of the brief assessment of cognition in schizophrenia; BCIS, beck cognitive insight scale; BDII, bipolar disorder type II; BP, bipolar depression; CAARS-Inv, Conner’ adult ADHD rating scale-investigator rated; ChSZ, chronic schizophrenia; CON, healthy control or typically developing group; DLPFC, dorsolateral prefrontal cortex; IQ, intelligence quotient; FDR, false discovery rate; FEP, first-episode psychosis; FPC, frontal pole cortex; HAM-D, Hamilton Rating Scale-Revised; HFA, high functioning autism; MDD, major depressive disorder; MPFC, medial prefrontal cortex; PANAS, Positive and Negative Syndrome Scale; PFC, prefrontal cortex; SASS, Social Adaptation Self-evaluation Scale; SCSQ, Social Cognition Screening Questionnaire; SCZ, schizophrenia; ToM, theory of mind; UHR, ultra-high-risk; VFT, Verbal Fluency Test; VLPFC, ventrolateral prefrontal cortex; YMRS, Young Mania Rating Scale.*

Patients with various psychiatric disorders typically have executive dysfunction and frequently demonstrate hypoactivation of the LPFC relative to healthy controls during executive tasks, such as the n-back task, go/no-go task, trail making test, and Stroop task. This has been observed using NIRS (Table 3) in addition to fMRI or PET studies [meta-analysis of schizophrenia (Minzenberg et al., 2009), major depressive disorder (MDD) (Snyder, 2013), and various participants (McTeague et al., 2017)]. In such cases, activation of the LPFC region serves as an index for successful psychotherapy. Cognitive remediation therapy has been found to increase DLPFC activity during n-back tasks in patients with schizophrenia, as assessed using NIRS (Pu et al., 2014) and fMRI (Haut et al., 2010).

In contrast, hyperactivation of the LPFC during executive tasks may be observed in some psychiatric patients. Firstly, patients with an anxiety disorder or post-traumatic disorder (PTSD) have been found to show hyperactivation of the LPFC when fearful or under stress (Ohtani et al., 2009; Rosenbaum et al., 2020b; Tables 1, 2). It has been suggested that this is because these individuals must exert significant effort to inhibit excessive emotion. In such cases, deactivation of the LPFC region is an index for improvement (Ohtani et al., 2009; Okamoto et al., 2010; Amano and Toichi, 2016; Glassman et al., 2016; Ong et al., 2020; Rosenbaum et al., 2020a,b; Table 2). Implementation of exposure therapy reduced the LPFC activity while watching videos of spiders in patients with arachnophobia (Rosenbaum et al., 2020b; Tables 1, 2). Similarly, eye movement desensitization and reprocessing have been found to reduce LPFC activity during the recall of traumatic memories (Ohtani et al., 2009; Tables 1, 2). Secondly, patients with ADHD may sometimes show increased LPFC activity during demanding inhibitory tasks such as Stroop tasks (Tsujimoto et al., 2013; Suzuki et al., 2017; Table 3). It has been suggested that this may occur because of a compensatory mechanism; when a task is demanding with high difficulty, increased efforts are required, leading to excessive brain activity (Tsujimoto et al., 2013). The compensatory mechanism has been also indicated in patients with schizophrenia (Koike et al., 2011; Nakano et al., 2018), bipolar disorder (Ono et al., 2017), and ecstasy polydrug users (Roberts and Montgomery, 2015) when tasks are demanding or symptoms are severe. Nevertheless, it has been suggested that this phenomenon can be distinguished by the lack of associated behavioral differences (Roberts and Montgomery, 2015). Finally, increased PFC activity may also be observed due to excessive cognitive processing caused by impaired selective attention or dysfunction of the default mode network (Ikeda et al., 2013). Patients with MDD showed increased bilateral PFC activity during Stroop tasks (Ikeda et al., 2013). However, activation attributed to this factor is more likely associated with the MPFC rather than with the LPFC considering that the MPFC is a core region of the default mode network (e.g., Gusnard and Raichle, 2001; Gusnard et al., 2001).

With regard to task selection, typical executive tasks that are robust and relatively consistent would be appropriate for clinical assessment. Habituation (e.g., Thompson, 2009), in such forms as a response decrement in brain activity as reported in fMRI studies (e.g., Breiter et al., 1996; Wright et al., 2001), can be caused by repetitive implementation of tasks, especially for multi-session interventions. Considering the basic properties of habituation (Thompson, 2009), this may be reduced by the following efforts. Task difficulty, which can be initially adjusted for each individual, should not be too easy, considering that a weaker stimulus is likely to cause habituation. The frequency of measurements can be decreased by omitting them at some sessions. Alternatively, it is also possible that tasks that differ from the assessment task are administered as dummy tasks to avoid monotony caused by repetition of a single type of task, considering that presentation of another stimulus may prevent or provide recovery from habituation to target stimuli. Moreover, use of a control task (e.g., 1-back task for n-back) for baseline data instead of rest may eliminate confounding (habituation) effects that can be caused by sensory adaptation (e.g., visual and tactile sensations) given that differences in rCBF changes between control and experimental tasks may computationally exclude the confounding effects due to sensory adaptation.

Finally, although these NIRS indexes can be used for the evaluation of psychotherapeutic effects in general research, it is preferable that they are used in addition to existing methods. The validity of NIRS indexes need to be examined using multiple lines of indexes from existing methods. Since these indexes assess different aspects, they may show inconsistency. For example, changes in brain activity can be detected without changes in self-report measures and vice versa. However, a holistic evaluation would provide information that can more thoroughly examine the results.

### Considerations for the Use of NIRS as a Practical Clinical Instrument

Near-infrared spectroscopy indexes can be used for the evaluation of psychotherapeutic effects within research settings; however, they are not yet applicable for routine clinical practice at the present stage. The applicability or usefulness of biomarkers (such as neuroimaging) may differ between the clinical research framework and core clinical framework (Albert et al., 2011), and translating them to routine clinical practices would still be premature (Dubois et al., 2014; Jack et al., 2018; Canevelli et al., 2019).

The above discussion indicates that methods with high levels of correlation between the brain and behavior with respect to psychiatric diagnoses (e.g., depression) are currently insufficient, and their relationship remains to be well characterized. Moreover, conventional methods, such as self-report and clinical data (e.g., behavioral data), are more strongly correlated with clinical markers (e.g., relapse, life-satisfaction, mortality, and suicide). Although there is neuroimaging evidence on psychotherapeutic effects, these results were obtained in a research setting rather than in routine clinical practice.

In order to translate the NIRS method to routine clinical practice, there needs to be adequate research results showing that NIRS is not harmful, and that it is reliable, valid, and beneficial to existing methods. Moreover, significant and important decisions in actual clinical practice and research settings (e.g., treatment choices) need to be based on adequate evidence from existing methods. In the field of psychiatric disorders, these aforementioned criteria remain unmet, and the validity of NIRS methods has not been adequately researched.

Nevertheless, studies using NIRS to investigate psychotherapeutic effects has started to increase in recent years, as shown in this systematic search (Tables 1, 2). Most of these studies have found psychotherapeutic effects based on changes in hemodynamic responses. Furthermore, the results of the systematic search show that the signs of improvement, indicated by the NIRS index, are relatively consistent with data shown with the behavioral or symptomatic changes (Table 2). Statistically significant correlations between the NIRS index and the behavioral or demographic data have also been frequently found, as shown in Tables 2, 3. Although these findings were obtained within a research framework, they indicate that the reliability and validity of NIRS to evaluate psychotherapeutic effects have been increasing recently. Although this field is still in an early developmental stage, it has great potential to grow in the future.

## Limitations and Possibilities

First, the pre- and post-intervention measurements interposed in a single session capture state changes in brain activity rather than trait changes. Hence, observed changes may be transient. It is unclear whether repeated state changes induced by multi-session implementations could lead to the trait changes that are usually considered as the outcome of psychotherapy. Measurements in multi-sessions should be able to address this question. Second, multi-session evaluations may be affected by changes in the patient’s or client’s mental and physical conditions. For example, naturalistic negative mood was found to correlate with lower activation in the DLPFC region during verbal working memory tasks in healthy participants (Aoki et al., 2011; Sato et al., 2014). However, the relationship between mental and physical conditions and rCBF changes in a single session can be examined by employing other assessment methods (e.g., self-report and interview). Third, NIRS application can also be beneficial in fields other than clinical psychology, including psychiatry and neurology. The effects of treatment methods, such as the repetitive transcranial magnetic stimulation and the transcranial direct-current stimulation, can be evaluated using methodologies similar with the ones discussed in this article. The use of NIRS as brain-machine interface also has high potential as an intervention tool for certain neurological (e.g., locked-in syndrome; Hong et al., 2018) and psychiatric symptoms (e.g., ADHD symptoms with NIRS-neurorehabilitation; Hudak et al., 2017; Kimmig et al., 2019). Fourth, due to the limited number of used search words and databases in the systematic search, Tables 1, 2 include only limited number of available studies which examined psychotherapeutic effects using NIRS; however, they provided information that helped the organization of the primary methodologies. Fifth, NIRS application can be useful for various psychotherapies, including ones not found in the current search results. Particularly, single- and multi-session evaluations using NIRS may be beneficial when other methodologies are less likely to be undertaken. For example, few methodologies may be available for some psychotherapeutic interventions, such as play therapy, sand therapy, and art-related therapy, compared to other psychotherapeutic approaches, such as cognitive behavioral therapy. Further, compared to adults without disorders, the available methodologies for children and some patients with difficulties in grasping their mental and physical conditions are limited. These issues are related to methodological availability. Single- and multi-session evaluations using NIRS provide another opportunity for such cases and may show brain-based evidence. Finally, this article provides a conceptual schema for the evaluation of psychotherapeutic effects using NIRS. Therefore, the suggested methodology and the NIRS index remain only a possibility. More detailed examination based on each psychiatric disorder and type of psychotherapy is warranted in future studies to approach actualization of evidence-based psychotherapy.

## Conclusion

This article highlights the possibility of using NIRS for evidence-based psychotherapy as a future direction in clinical psychology, it discussed some of the benefits and challenges of applying this technique for this purpose, and organized the available methodology based on a review of primary studies that have used NIRS to examine psychotherapeutic effects. Furthermore, this perspective article proposed that single- and multi-session evaluations with NIRS as alternative methodologies for the evaluation of psychotherapeutic effects. Since these methods capture state changes in brain activity, immediate measurements are important. Changes in oxyHb levels of the LPFC region during typical executive tasks were suggested as potential NIRS indexes. Although more detailed examination is needed in future studies, single- and multi-session evaluations using NIRS may provide additional information which can be derived from psychotherapeutic interventions.

## Author Contributions

SO provided all perspectives and prepared the manuscript.

## Conflict of Interest

The author declares that the research was conducted in the absence of any commercial or financial relationships that could be construed as a potential conflict of interest.

## Publisher’s Note

All claims expressed in this article are solely those of the authors and do not necessarily represent those of their affiliated organizations, or those of the publisher, the editors and the reviewers. Any product that may be evaluated in this article, or claim that may be made by its manufacturer, is not guaranteed or endorsed by the publisher.
